# Acoustic Realism of Clinical Speech-in-Noise Testing: Parameter Ranges of Speech-Likeness, Interaural Coherence, and Interaural Differences

**DOI:** 10.1177/23312165251336625

**Published:** 2025-05-06

**Authors:** S Theo Goverts, Virginia Best, Julia Bouwmeester, Cas Smits, H Steven Colburn

**Affiliations:** 1 Department of Otolaryngology-Head and Neck Surgery, Section Ear & Hearing, Amsterdam Public Health Research Institute, Amsterdam UMC Location Vrije Universiteit, Amsterdam, the Netherlands; 2 Department of Speech, Language and Hearing Sciences, 1846 Boston University, Boston, MA, USA; 3 Department of Otolaryngology-Head and Neck Surgery, Amsterdam Public Health Research Institute, Amsterdam UMC Location University of Amsterdam, Amsterdam, the Netherlands; 4 Department of Biomedical Engineering, 1846 Boston University, Boston, MA, USA

**Keywords:** hearing assessment, ecological validity, acoustic realism, binaural recordings, natural environments

## Abstract

Speech-in-noise testing is a valuable component of audiological examination that can provide estimates of a listener's ability to communicate in their everyday life. It has long been recognized, however, that the acoustics of real-world environments are complex and variable and not well represented by a typical clinical test setup. The first aim of this study was to quantify real-world environments in terms of several acoustic parameters that may be relevant for speech understanding (namely speech-likeness, interaural coherence, and interaural time and level differences). Earlier acoustic analyses of binaural recordings in natural environments were extended to binaural re-creations of natural environments that included conversational speech embedded in recorded backgrounds and allowed a systematic manipulation of signal-to-noise ratio. The second aim of the study was to examine these same parameters in typical clinical speech-in-noise tests and consider the “acoustic realism” of such tests. We confirmed that the parameter spaces of natural environments are poorly covered by those of the most commonly used clinical test with one frontal loudspeaker. We also demonstrated that a simple variation of the clinical test, which uses two spatially separated loudspeakers to present speech and noise, leads to better coverage of the parameter spaces of natural environments. Overall, the results provide a framework for characterizing different listening environments that may guide future efforts to increase the real-world relevance of clinical speech-in-noise testing.

## Introduction

The goal of auditory rehabilitation is to improve functioning in everyday life for individuals who live with hearing loss. To diagnose hearing loss, and to prescribe and assess the success of auditory rehabilitation, clinicians often rely on behavioral tests of functional auditory abilities (such as speech understanding) using the available clinical infrastructure. It has long been recognized that clinical hearing tests are a simplification of real-world listening situations, and that this may explain why clinical measures sometimes do not show good correspondence with a patient's real-world experience (e.g., Badajoz-Davila & Buchholz, 2021; Cord et al., 2004; Gnewikow et al., 2009). To combat this disconnect, there is a lot of interest in developing clinical tests that are more relevant or have higher “ecological validity” (e.g., Keidser et al., 2020). It should be noted that this term actually merges two related but distinct elements: realism and external validity (Beechey, 2022; Keidser et al., 2020, 2022). Realism refers to the similarity of relevant parameters in the test setting to those in natural situations, whereas external validity refers to the correspondence between the results of a certain test and performance in natural situations.

For speech-in-noise (SIN) testing, several broad domains have been identified that may modulate ecological validity, including the linguistic content of the speech stimuli (e.g., Best et al., 2016); the social context in which stimuli are presented (e.g., Carlile & Keidser, 2020); and the cognitive demands of a particular task (e.g., Lunner et al., 2020). In the current study we focus on a domain that we call “acoustic realism”, which motivates our own investigations and has received increasing attention in the literature in recent years. For example, several studies have aimed to measure the range of realistic signal-to-noise ratios (SNRs) that listeners encounter when communicating in their daily life (e.g., Mansour et al., 2021a; Smeds et al., 2015; Weisser & Buchholz, 2019; Wu et al., 2018) and have argued that SIN testing that targets these SNRs may lead to more relevant outcome measures. Other studies have investigated the consequences of introducing more realistic background noises into SIN tests (e.g., Best et al., 2015; Mansour et al., 2021b), considering factors such as amplitude modulation, informational masking, spatialization, and reverberation. These studies demonstrate that variations in realism can affect outcome measures and even the conclusions one might draw from SIN testing.

Goverts and Colburn (2020) approached acoustic realism by documenting the acoustics of natural environments. They obtained binaural recordings from the ears of human subjects actively immersed in eight everyday environments (Home, Restaurant, City Walk, City Talk, City Bike Ride, Station, Train, and Bus). From those recordings, they extracted a number of acoustic parameters that related to the spatial characteristics of the natural environments, including interaural coherence (IC), interaural time differences (ITDs), and interaural level differences (ILDs). This analysis showed that natural environments result in a wide range of interaural parameters as measured at the ears, but also that only a small proportion of measurements show high IC. This implies that highly reliable information in realistic scenarios is relatively sparse, and indeed there are many demonstrations in the literature of binaural mechanisms designed to take advantage of this fact (e.g., Dietz et al., 2013; Stecker et al., 2021). Goverts and Colburn also quantified “speech-likeness” (SpLi) at the left and right ears, using a modulation-spectrum-based metric for which high values indicate a high similarity to speech. This metric provides a convenient estimate of the presence of speech in a noisy signal, and is related to the SNR, but can be calculated non-intrusively (i.e., without access to the signal and the noise separately). SpLi results showed large variability within and between environments, and revealed prevalent across-ear asymmetries, highlighting that there is often an acoustically better ear for speech reception that can change from moment to moment.

In the current study, our first goal was to further characterize the acoustic properties of real-world environments, especially those in which people typically communicate. The approach closely followed that of Goverts and Colburn (2020), but provided additional insights. For example, because the recordings of Goverts and Colburn (2020) were conducted in real-world settings, they contained speech and other sounds in rather uncontrolled combinations. In the current study, we used realistic simulations that afforded us much more control. Specifically, we chose an existing set of recordings of six everyday environments in which communication often takes place (Office, Church, Living Room, Café, Dinner Party, and Food Court), and combined them with conversational speech materials that are intended for those environments. As the waveforms for both environments and conversational sentences were available separately, it was possible to vary the SNR, and thus calculate SpLi, IC, ITD and ILD as a function of SNR.

The second goal of the study was to consider and quantify the acoustic realism of clinical SIN testing using the same analyses we applied to the real-world environments. It is important to point out that SIN testing is not consistently included as a part of regular clinical audiological care (e.g., Billings et al., 2024; Fitzgerald et al., 2023; Parmar et al., 2022; Vermiglio et al., 2019). When it is included, there are variations in its format across different clinics and countries. SIN testing may be performed over headphones to assess ear-specific impacts of hearing loss, or it may be performed using an external loudspeaker to assess a patient's functional speech recognition capacity with or without hearing-assistive devices. In the latter case, a typical test setup uses co-located speech and noise stimuli played from a single loudspeaker. Arrangements using two or more loudspeakers are common in clinical research studies (e.g., Corbin et al., 2021; D’Onofrio et al., 2020; Griffin et al., 2019; Vermeire & Van de Heyning, 2009) and in certain clinical settings for sound localization testing (e.g., Ramirez et al., 2024) but to date such setups are not in widespread use for SIN testing in clinical care. Beyond the physical test setup, there are also many variations in the stimuli that may be considered for SIN testing. Target stimuli may be words, digit triplets, sentences, or stories (Griffin et al., 2020; Kollmeier et al., 2015; Nilsson et al., 1994; Plomp & Mimpen, 1979; Smits et al., 2013; Versfeld et al., 2000; Wilson et al., 2007; see also reviews in Billings et al., 2024 and Sanchez et al., 2022). The interfering signal is typically a stationary speech-spectrum noise, but may also be fluctuating noise, babble, reversed speech, or speech (e.g., Versfeld et al., 2021; Wilson et al., 2007). Most commonly, SIN performance is quantified according to an adaptive procedure that estimates the SNR where 50% of the speech items are recognized correctly (Levitt, 1971; Nilsson et al., 1994; Plomp & Mimpen, 1979). Variants of test procedure use different target points in the adaptive procedure, or use fixed SNRs (e.g., Dambha et al., 2022; Herbert et al., 2023).

In the current study we took one example of a clinical SIN test, which was the basic single-loudspeaker test used in Dutch audiology clinics (with the loudspeaker at 0° azimuth). This test uses sentences embedded in stationary speech shaped noise, and an adaptive procedure targeting 50%. We made binaural recordings using microphones placed in the ears of a listener completing this test, to facilitate a direct comparison with the real-world recordings and simulations described above. Our aim here was to quantify the parameter spaces for the clinical test and understand which parameters deviate most from the real-world environments. In addition, we wanted to understand how much these parameter spaces change (or how much acoustic realism may be improved) by simply adding a second loudspeaker to the setup. To that end, we also implemented and made recordings during a simple two-loudspeaker variation of the Dutch test with the speech and noise at ±45° azimuth. We chose a two-loudspeaker version deliberately given that it is a highly feasible arrangement to set up in a typical clinic; most audiometric booths are large enough to house two loudspeakers in this configuration and have two audio channels readily available.

To address these goals, the approach of the present study was to:

1. Extend the analyses of SpLi, IC, ITD, and ILD from Goverts and Colburn (2020) to six realistic communication-focused environments and to binaural recordings of two clinical SIN tests;
2. Explore the effect of SNR on SpLi, IC, ITD, and ILD in the communication-focused environments;
3. Quantitatively compare parameter spaces of SpLi, IC, ITD, and ILD for the realistic environments and for the clinical SIN tests.

## Methods

### Binaural Stimuli

#### Binaural Recordings of Natural Environments (“Binaural Recordings”)

Binaural recordings made in eight natural environments were used as described in Goverts and Colburn (2020). The purpose of that earlier study was to have a representative, though far from complete, set of environments that individuals with hearing loss are active in. The choice of the specific environments was inspired by interviews with specialists involved in research or in clinical care or who live with hearing loss. The environments included Home, Restaurant, City Walk, City Talk, City Bike Ride, Station, Train, and Bus. In-ear recordings were made using two commercially available microphones (Sound Professionals MS-TFB-2) and a digital recorder (Olympus linear PCM recorder, Model LS-11) at a 44.1-kHz sample rate. The microphones were placed in the concha. Note that the subjects who wore the microphones in their ears were not just observers but actively participated in the environments.

#### Binaural Re-Creations of Communication Situations (“Binaural Re-Creations”)

The binaural re-creations made use of six natural environments from the Ambisonic Recordings of Typical Environments (ARTE) database (Weisser et al., 2019). These environments included Office, Church, Living room, Café, Dinner Party, and Food Court. The ARTE database contains 64-channel recordings in the environments that were converted for binaural playback as described by Weisser et al. (2019). For each environment, those recordings can be combined with “clean” sentences extracted from conversations held by real talkers conversing while listening to the recordings over headphones (see Miles et al., 2020). We followed the approach of Miles et al. (2022), which was to spatialize the sentences using head-related transfer functions measured in each environment using an acoustic manikin and a source at a distance of 1 m directly in front. A unique realistic SNR was chosen for each environment (−6.6, −3.0, −1.3, 1.6, 3.0, 6.2 dB for the Office, Church, Living Room, Café, Dinner Party, and Food Court, respectively) based on the estimates made by Weisser and Buchholz (2019). The analyses presented in the current study made use of 4 lists of 16 target sentences spoken by a single female talker (see Miles et al., 2020). Furthermore, for each environment, mixtures were generated with SNRs varying from −30 dB through +30 dB in 5 dB steps. We note that the binaural stimuli in the condition with SNR of −30 dB are dominated by the background sounds, whereas the binaural stimuli in the condition with SNR of +30 dB are dominated by the conversational speech.

#### Binaural Recordings of “Clinical SIN Tests”

Using the same setup described above, binaural recordings were made in an audiological clinic at Amsterdam UMC during the administration of two SIN tests using the stimuli of Versfeld et al. (2000). The first was the commonly used test in which the target speech (Dutch sentences spoken by a female talker) and the steady state masking noise (female Long-Term Average Speech Shaped noise; LTASS) are co-located, presented via one loudspeaker at approximately 1.5 m in front of the listener. This test is referred to as the “clinical SIN test.” The second test was a two-loudspeaker variation based on the test described by Boymans et al. (2009). The test uses two loudspeakers positioned at ±45° at 1.5 m distance from the listener, which present the target speech and masking noise. In our variation, two adaptive tracks were interleaved. One corresponded to a female talker on the right presented with steady state male LTASS noise on the left, and the other corresponded to a male talker on the left presented with steady state female LTASS noise on the right. This test is referred to as the “spatialized clinical SIN test.” A volunteer with normal hearing completed two runs of each of these two tests. Initially, the regular adaptive procedure was followed, with the listener providing verbal responses and all responses (correct/incorrect) were noted. The actual recording was made in the second run, where we replicated this procedure, but without the listener responding, to avoid the listener's own voice appearing in the recordings and influencing the acoustic analyses. The listener was instructed to make no head movements, though the head was not fixed.

### Calculation of Acoustic Parameters

SpLi was calculated using the approach of Goverts and Colburn (2020), and further details can be found there. Briefly, signals were processed into non-overlapping 1-second time-slices (with 0.5-seconds cosine-shaped rise and fall times) and bandpass filters were applied resulting in six, one-octave frequency bands centered at 250, 500, 1,000, 2,000, 4,000, and 8,000 Hz. For each frequency band the envelope was extracted and the discrete Fourier transform of this envelope was calculated. The modulation energy for each frequency band was determined in each of the six one-octave modulation bands centered at 4, 8, 16, 32, 64, and 128 Hz. Then for each spectral band the speech-to-reverberation modulation energy ratio (SRMR) was calculated as the ratio between the sum of energies present at low modulation frequencies and the sum of energies present at high modulation frequencies. This modulation-strength measure was developed and used by Falk and colleagues (e.g., Cosentino et al., 2014; Falk et al., 2010) in their studies of speech quality and intelligibility. It is based on the assumption that low modulation frequencies are related primarily to speech, and high modulation frequencies are related primarily to noise or reverberation. Goverts and Colburn (2020) adapted this measure to estimate SpLi in dB, SpLi(*f_i_*) = 10 × log_10_(SRMR(*f_i_*)), and then calculated a broadband SpLi using a speech-intelligibility-index-weighted sum of the SpLi(*f_i_*) values. The resulting SpLi values vary from about −2 dB for a stationary masker to 10 dB for natural clean speech and 12.5 dB for well-pronounced clean recorded speech materials used in SIN tests (Goverts & Colburn, 2020). SpLi was calculated for both left and right ears.

The interaural parameters were also calculated using the approach of Goverts and Colburn (2020), and further details can be found there. The left and right signals were filtered through a bank of quarter-octave-bandwidth filters, and selected frequencies were analyzed with respect to their interaural differences. In the results reported here, following Goverts and Colburn (2020), one low-frequency band (centered at 500 Hz) and one higher frequency band (centered at 2,000 Hz) were chosen. For the band at 2,000 Hz, only the interaural information contained within the envelopes of the 2,000-Hz waveforms was examined. In all cases, the bilateral signals were analyzed in 300-ms time-slices including with 5-ms, cosine-shaped rise and fall times. Successive analysis windows were shifted by 20-ms and thus partially overlapping. The window duration was somewhat arbitrary, but followed that used in Goverts and Colburn (2020) and was broadly in line with window sizes used in previous studies that have considered spectrotemporal “glimpses” in speech (e.g., Conroy et al., 2020) or have estimated binaural integration windows under different conditions (e.g., Dietz et al., 2013; Eurich & Dietz, 2023; Reed et al., 2016).

For each time window, we computed the cross-correlation function using the left and right waveforms. The cross-correlation was normalized by the product of the root-mean-square amplitudes, giving a maximum value of unity for waveforms that differ only in a pure delay and/or a fixed scale factor. The IC was defined as the maximum normalized correlation. The ITD was defined as the time shift for which the cross-correlation function reached its maximum value. Windows with an ITD larger than 1 ms were not included in the ITD distributions. Moreover, if the IC for a given window was less than 0.5, then that window was not included in the ITD distributions. The ILD was defined as the difference in dB between the two ears for each window. Whereas Goverts and Colburn (2020) analyzed IC, ITD and ILD in both 500 and 2,000 Hz bands, in the current study we considered only the IC and ITD at 500 Hz, and the ILD at 2,000 Hz, according to the frequency range generally considered to be most relevant for each kind of cue (e.g., Hartmann, 2021).

## Results

### Parameter Spaces of Speech-Likeness

**Figure 1.**
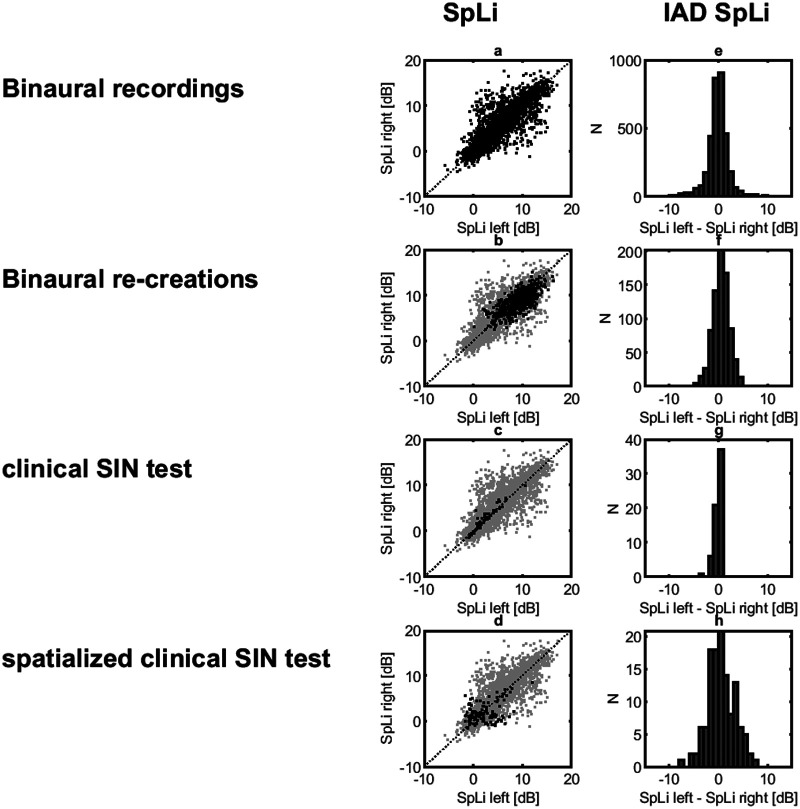
Parameter Spaces and Left–Right Asymmetries for SpLi. The Rows Present Analyses of Binaural Recordings in Eight Natural Environments (Panels a, e), in the Binaural Re-creations (Panels b, f), During the Clinical SIN Test (Panels c, g) and During the Spatialized Clinical SIN Test (Panels d, h). The First Column Presents Parameter Spaces for SpLi. In Each Panel, SpLi Values for the Right Ear are Plotted Against SpLi Values for the Left Ear. The Distribution from Panel a is Plotted in Gray in the Other Panels to Facilitate Comparisons to the Earlier Study. The Second Column Presents Interaural SpLi Differences to Illustrate the Amount of Asymmetry.

Figure 1 displays the parameter spaces and left–right asymmetries for SpLi. The rows present results for the binaural recordings in the eight natural environments (panels a, e), the binaural re-recreations at their realistic SNRs (panels b, f), the clinical SIN test (panels c, g), and the spatialized clinical SIN test (panels d, h). In the first column SpLi values for the right ear are plotted against SpLi values for the left ear (in black). For reference, the parameter spaces for the binaural recordings of panel a are replotted in gray in the other panels. For the binaural recordings, the SpLi values range from −6 to 18 dB. For the binaural re-creations, the distribution of SpLi values was similar but with a slightly reduced range (0–16 dB); the absence of extremely low values in the binaural re-creations is because speech was always present and at a sufficiently high SNR to support communication. The clinical SIN test shows a restricted distribution of SpLi values that is weighted towards low values (−1 to 11 dB) and includes very few asymmetries. The spatialized clinical SIN test has a similar range (−2 to 12 dB) but shows more asymmetry as a result of the laterally placed targets and maskers. The overall low values of SpLi observed in both versions of the clinical test reflect the dominance of negative SNRs in the adaptive tracking procedure, as is typically observed. The second column of Figure 1 displays histograms of the left–right ear asymmetries in SpLi. This representation shows that the distribution of asymmetries is comparable across the binaural recordings and the binaural re-creations. The clinical SIN test has a very tight distribution close to zero, but the spatialized version has a broader distribution comparable to that of the natural environments. Statistical testing (*F* tests with Bonferroni correction) showed significant differences between all distributions (*P* < .01).

### Parameter Spaces of Interaural Coherence, Interaural Time Differences, and Interaural Level Differences

**Figure 2.**
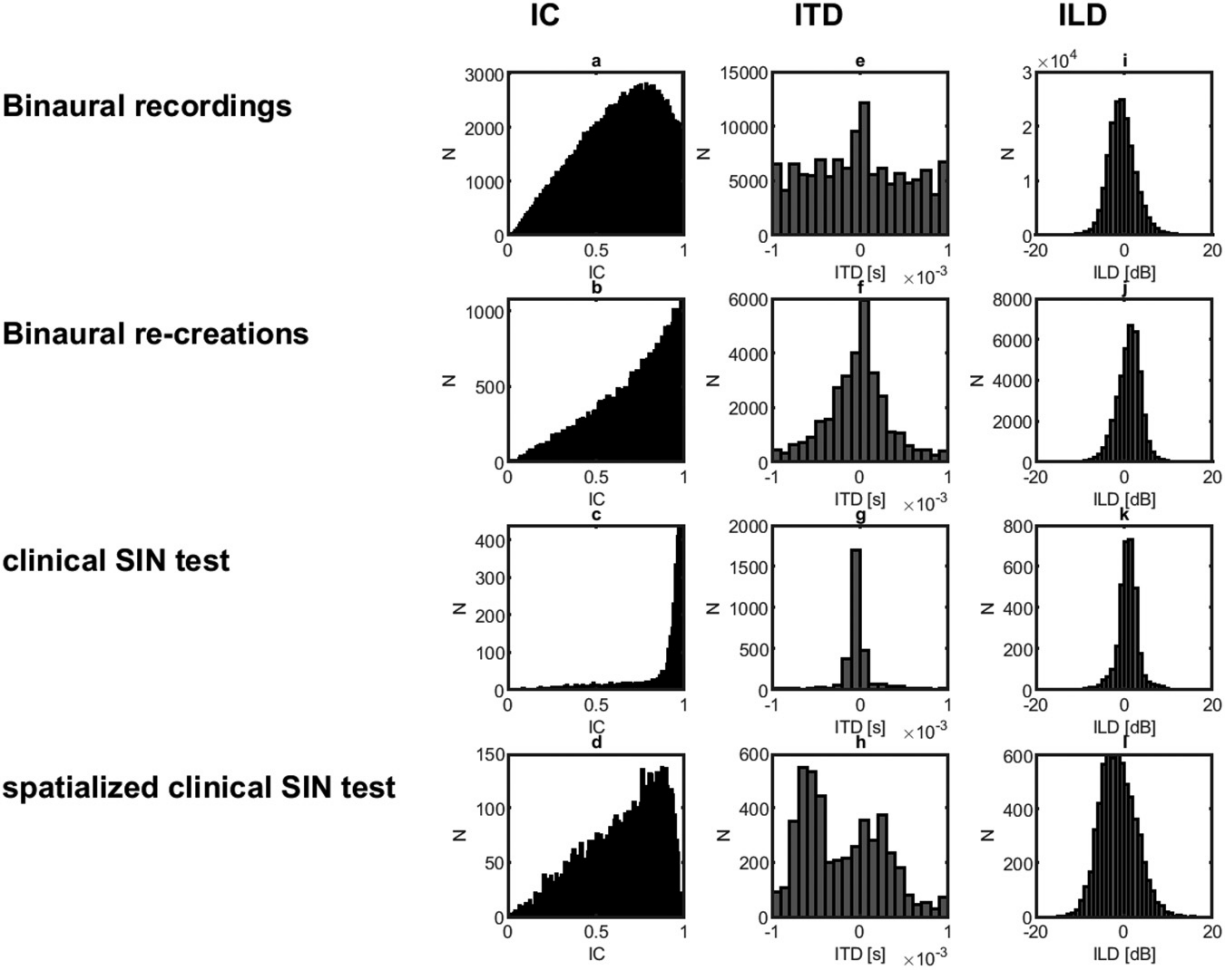
Parameter Spaces for the IC at 500 Hz, ITD at 500 Hz and ILD at 2,000 Hz. The Rows Present Analyses of Binaural Recordings in Eight Natural Environments (Panels a, e, i), in the Binaural Re-creations (Panels b, f, j), During the Clinical SIN Test (Panels c, g, k) and During the Spatialized Clinical SIN Test (Panels d, h, l). Histograms of IC Values, ITD Values, and ILD Values are Presented in the First, Second, and Third Column, Respectively.

Figure 2 displays the parameter spaces for the IC at 500 Hz, ITD at 500 Hz, and ILD 2,000 Hz in the first, second and third column, respectively. Comparing panels a and b, it appears that the binaural recordings, which were collected during a variety of activities, have a stronger representation of low IC values than the binaural re-creations. The effect of the frontal target talker at a relatively close distance in the binaural re-creations gives a shift towards high IC values, although the binaural recordings include segments with the microphone wearer's own voice, which likely explains the strong representation of IC values at 1 (Pohlhausen et al., 2022). The clinical SIN test shows a highly skewed distribution of IC values near unity correlation (panel c), whereas the distribution for the spatialized clinical SIN test (panel d) has a broader distribution that much more closely matches that of the natural environments. Statistical testing (*F* tests with Bonferroni correction) showed significant differences between all distributions (*P* < .01), except for the difference between the binaural re-creations and the spatialized clinical test (*P* = .24). The second column of Figure 2 displays the parameter spaces for the ITD at 500 Hz. Comparing panels e and f, it appears that the binaural recordings have a stronger representation of extreme ITDs than the binaural re-creations. The effect of the frontal target talker at a relatively close distance in the binaural re-creations is visible in stronger representation of ITD values around 0 ms. The clinical SIN test shows an even stronger representation of ITD values near 0 ms (panel g), whereas the distribution for the spatialized clinical SIN test (panel h) has a range of values that more closely matches that of the natural environments. Statistical testing (*F* tests with Bonferroni correction) showed significant differences between all distributions (*P* < .01), except for the difference between the binaural re-creations and the spatialized clinical test (*P* = .013).

The third column of Figure 2 displays the parameter spaces for the ILD at 2,000 Hz. Comparing panels i and j, it appears that the different sets of binaural recordings have a similar distribution of ILD. The effect of the frontal target talker at a relatively close distance in the binaural re-creations is partly visible in a more centrally weighted representation. The clinical SIN test also shows a representation of ILD values that is focused around 0 dB (panel k), whereas the distribution for the spatialized clinical SIN test (panel l) has a broader distribution that is even broader than that of the natural recordings and the binaural re-creations. Statistical testing (*F* tests with Bonferroni correction) showed significant differences between all distributions (*P* < .01).

### Parameter Variations as a Function of SNR

**Figure 3.**
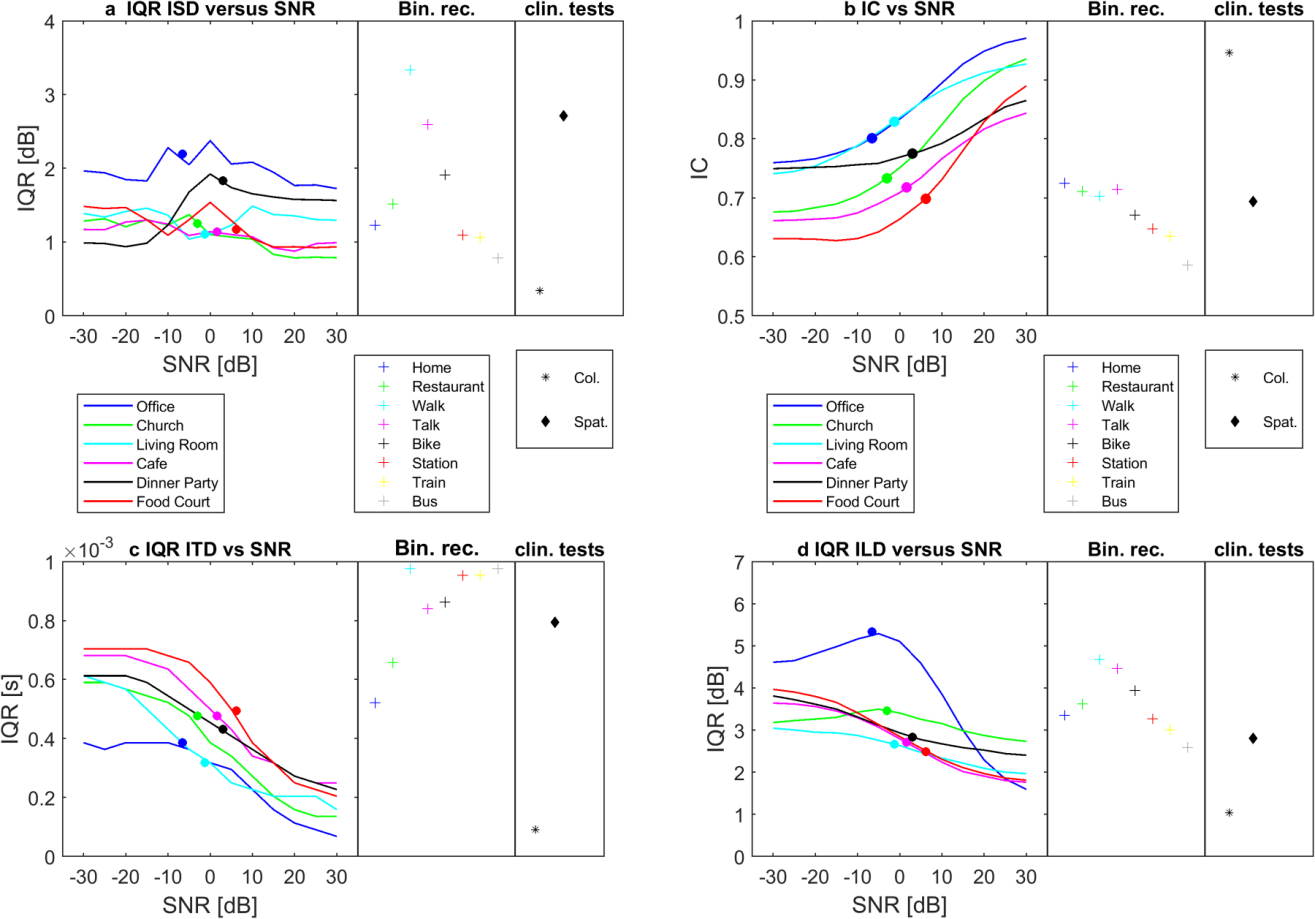
Key Interaural Measures as a Function of SNR for the Binaural Re-creations. For each environment, the value of the Interaural Measures at its specific ecological SNR is indicated by a colored dot. For Comparison, on the Right Side of Each Panel, Values for the Binaural Recordings and the Two Clinical SIN Tests are Presented. Shown are Interquartile Ranges of the Interaural SpLi Differences (IQR-ISD; Panel a), Median Values of IC (Panel b), Interquartile Ranges of ITDs (IQR-ITD; Panel c), and Interquartile Ranges of ILDs (IQR-ILD, panel d).

Figure 3 presents several key interaural measures as a function of SNR for the binaural re-creations. For comparison, on the right side of each panel, values for the binaural recordings and the two clinical SIN tests are presented. Figure 3a shows interquartile ranges of the interaural SpLi differences (IQR-ISD), capturing the width of the distributions shown in Figure 1e–h. There seems to be no general pattern of dependence of this parameter on SNR that holds across all environments. For most of the binaural recordings, the IQR-ISD agrees well with that of the binaural re-creations at their ecological SNRs, although the City Walk and City Talk show rather high values, consistent with the fact that these are environments with dominant talkers to one side. The clinical SIN test shows a very reduced IQR-ISD, whereas the spatialized version produces rather high IQR-ISD values, in line with the more asymmetric binaural recordings. Figure 3b shows median values for IC, capturing the central tendency of the distributions shown in Figure 2a–d. The median IC shows a strong dependence on SNR for all environments. At the low-SNR end, the differences across environments reflect differences in the competing low-frequency sounds, their spatial configuration and the reverberation present. At the high-SNR end, the differences across environments reflect differences in how the acoustics of the room or environment impact the target talker. The highest median IC values, over the range of SNRs, are found for the Office and Living Room, whereas the lowest values are found for the Food Court and Café. For most of the binaural recordings the median IC agrees well with that of the binaural re-creations at their ecological SNRs, although the Station, Train, and Bus recordings show relatively low values. This reflects the fact that these are noisy environments in which communication was typically not occurring and there was no one dominant target source. The clinical SIN test shows very high IC values, corresponding to the binaural re-creations at +30 dB, whereas the spatialized version has a much-reduced median IC that matches well the IC for the binaural recordings and the binaural re-creations at their ecological SNRs. Figure 3c shows interquartile ranges of ITDs (IQR-ITD), capturing the width of the distributions shown in Figure 2e–h. The IQR-ITD shows a strong dependence on SNR for all environments. At the low-SNR end, the differences across environments reflect differences in the competing low-frequency sounds, their spatial configuration and the reverberation present. For most of the binaural recordings the IQR-ITDs are higher than those of the binaural re-creations, reflecting the fact that they are not always dominated by a centrally located talker. The clinical SIN test shows a very low IQR-ITD, similar to the binaural re-creations at +30 dB, whereas the spatialized version has an IQR-ITD that agrees rather well with the binaural recordings. Figure 3d shows interquartile ranges of ILDs (IQR-ILD), capturing the width of the distributions shown in Figure 2i–l. The IQR-ILD does not depend strongly on SNR. For most of the binaural recordings, the IQR-ILD agrees well with that of the binaural re-creations. Again, the City Walk and City Talk show relatively high IQR-ILDs consistent with strongly asymmetric sources. The clinical SIN test shows a very low IQR-ILD compared to that of the binaural recordings and binaural re-creations, even lower than the binaural re-creations at +30 dB SNR. The spatialized version has an IQR-ILD that agrees rather well with that of the binaural recordings and the binaural re-creations at their ecological SNRs.

## Discussion

The first major goal of the current study was to characterize the acoustic properties of real-world environments in which people typically communicate, with an emphasis on those properties that are relevant for binaural hearing. To do so we extended the results of Goverts and Colburn (2020), who analyzed a broad range of everyday environments, to binaural re-creations of natural environments that included conversational speech embedded in real-world backgrounds. Applying a similar analysis to the new binaural re-creations produced broadly consistent results, but some key differences were noted. In general, the range of SpLi values was smaller, and the range of interaural SpLi differences was smaller. Furthermore, the IC distribution for the re-creations had a greater fraction of higher values and the ITD and ILD ranges were smaller. These differences can largely be understood by the specific origin of the two sets of binaural stimuli. The binaural recordings cover a more diverse range of situations, including some that are not dominated by speech. Furthermore, in the binaural recordings the dominant source could move relative to the subject, leading to more/larger asymmetries than for the binaural re-creations, in which the dominant speech source was always close to and directly in front of the subject. Considering all of the binaural stimuli, everyday life can be considered as a condition with highly variable amounts of speech information, consistent with previous studies that estimated a wide range of SNRs in realistic environments (e.g., Mansour et al., 2021a; Smeds et al., 2015; Weisser & Buchholz, 2019; Wu et al., 2018). Our results also emphasize that everyday environments can be highly asymmetric, show interaural coherences varying from perfect (unity) to diffuse, and have binaural properties spanning a wide range of values.

One key advantage of the binaural re-creations over the real-world recordings is that they make it possible to directly explore the impact of SNR on the parameter spaces of communication-focused scenarios. Our data show that while many of the parameters we examined are sensitive to SNR, there are also differences in the parameters across environments for a given SNR. For example, the IC varies quite dramatically across environments, at a given SNR. The Office represents an environment with very little reverberation and showed a consistently high IC, suggesting that it offers good opportunities for extracting spatial information. The other environments are more reverberant and influence the IC in different ways depending on the size and characteristics of the room. For example, the Food Court is a large room with highly diffuse late reverberation that leads to low IC values particularly at low SNRs where the background sounds dominate. On the other hand, the Café is a small room with strong early reflections that interact with the target and reduce the IC even at the high-SNR end.

The second major goal of the current study was to measure the parameter spaces of clinical SIN tests in relation to realistic environments, to identify and quantify specific ways in which the clinical tests lack acoustic realism. For this comparison we considered both a typical single-loudspeaker test, which was expected to show very poor acoustic realism, but also explored how the picture changes for a simple variation on the clinical test, which uses two separated loudspeakers to present the speech and noise. We found that the parameter spaces of everyday environments are rather poorly covered by that of the clinical SIN test. The clinical SIN test includes only low SpLi values, which are only observed in the binaural re-creations for unnaturally low SNRs, or in the natural recordings that are not intended for speech communication. The clinical SIN test also displays very little asymmetry in SpLi, relatively limited variation in ITD and ILD, and very high IC values. Broadly speaking, the starkly different parameter spaces we observed for the clinical SIN test relative to the natural environments are not surprising and are well explained by the strong symmetry of the single-loudspeaker test. A concern for clinical practice is that this configuration produces performance measures that mainly reflect the capacities of the patient's better ear and will not reveal effects related to the poorer ear. This makes it difficult to assess symmetric losses, and may lead to over-estimates of real-world performance for this population (who may have to rely on their poorer ear in certain real-world situations). Moreover, the single-loudspeaker configuration does not require the utilization of binaural differences, and thus cannot reveal any specific deficits related to binaural processing (Bronkhorst & Plomp, 1990). Finally, the unnaturally high IC values in the clinical test avoid any assessment of the processing of stimuli with low IC. Thus, the clinical test may over-estimate how listeners will cope in real-world situations with multiple interferers and strong reverberation.

Encouragingly, we have demonstrated that it is relatively simple with one additional loudspeaker to introduce asymmetry, reduce IC, increase the range of ITDs and ILDs, and arrive at more realistic parameter spaces. The parameter spaces we observed for the spatialized clinical SIN test were generally well-matched to the parameter spaces of the realistic environments. Of course, other spatial configurations of a two-loudspeaker test will also lead to an improved coverage of the parameter spaces relative to the co-located clinical test, as will larger loudspeaker arrays. Moreover, there are certain characteristics of real-world environments that can only be captured with a larger number of sources. For example, a “symmetric” three-talker configuration is very commonly used to minimize acoustic effects related to the head shadow and focus instead on binaural processing (Cameron et al., 2009; Marrone et al., 2008; Srinivasan et al., 2020). Configurations with three or more talkers are also believed to maximize “informational masking” (Freyman et al., 2004; Simpson & Cooke, 2005) and thus capture the ability of listeners to overcome this particularly disruptive form of interference. However, the added value in terms of acoustic realism of adding a second loudspeaker to a one-loudspeaker setup is likely to be greater than going from two to three (or more) loudspeakers. From a practical perspective, two audio channels and two loudspeakers are relatively common in clinical settings and thus this configuration would be very feasible to implement widely in clinics (or countries) not currently doing so. It would be useful to make more extensive recordings of clinical tests with different loudspeaker configurations, materials, and hearing-impaired participants, to fully explore the range of parameter spaces that can be achieved.

Finally, it should be noted that, while we have explored just a few acoustic parameters, and focused on those relevant to binaural hearing, there are many more parameters that could be taken into account in the search for acoustic realism in clinical SIN testing (e.g., temporal dynamics and spectral characteristics of the background, variations in loudness, etc.). Furthermore, there are many non-acoustic factors that may affect the realism of a speech communication situation and even interact with the factors we have explored (e.g., visual information, linguistic content; see e.g., Keidser et al., 2020; Wu et al., 2018). An important next step is to determine the extent to which different deviations from realism have a meaningful impact on outcomes and on our ability to estimate an individual's functional abilities in the real world.

## Conclusions

Everyday situations in which people communicate vary widely in their binaural properties and are often highly asymmetric. These properties are not captured well by commonly used clinical speech tests that are limited to a single loudspeaker. Simple and clinically feasible test setups using as few as two loudspeakers can provide a much betterer coverage of the acoustic parameters encountered in daily life, which may be important for the assessment of functional SIN abilities, particularly in patients with asymmetric losses or binaural processing deficits.
